# Proteasome assembly chaperone translation upon stress requires Ede1 phase separation at the plasma membrane

**DOI:** 10.1016/j.isci.2023.108732

**Published:** 2023-12-14

**Authors:** Thomas D. Williams, Aurellia Winaya, Ifeoluwapo Joshua, Adrien Rousseau

**Affiliations:** 1 MRC-PPU, School of Life Sciences, University of Dundee, Dow Street, Dundee DD5 1EH, UK

**Keywords:** Molecular biology, Cell biology

## Abstract

Proteome adaptation is key to cells surviving stresses. Increased translation of proteasome assembly chaperones (PACs) is critical for increasing proteasome assembly and cell degradative capacity. The endocytic protein Ede1 recruits PAC mRNA to cortical actin patches in *Saccharomyces cerevisiae* for translation upon stress. We show, through genetic and pharmacological studies, that this is mediated by the capacity of Ede1 to phase separate. PAC expression is maintained when we exchange the phase separating domains from Ede1 for those of unrelated proteins. Without these phase separating regions, PAC expression is not induced upon stress, preventing increased proteasome assembly, causing cell death. This work identifies a mechanism underpinning Ede1-mediated increased translation of specific mRNAs at a time when general translation is repressed.

## Highlights

• Cells assemble more proteasomes upon stress to help them adapt and survive
• This requires increased proteasome assembly chaperones (PACs) translation
• Ede1 relocalizes PAC mRNA to cortical actin patches for translation upon stress
• Ede1 phase separation is essential for PAC mRNA relocalization

## Graphical abstract

**Figure:**
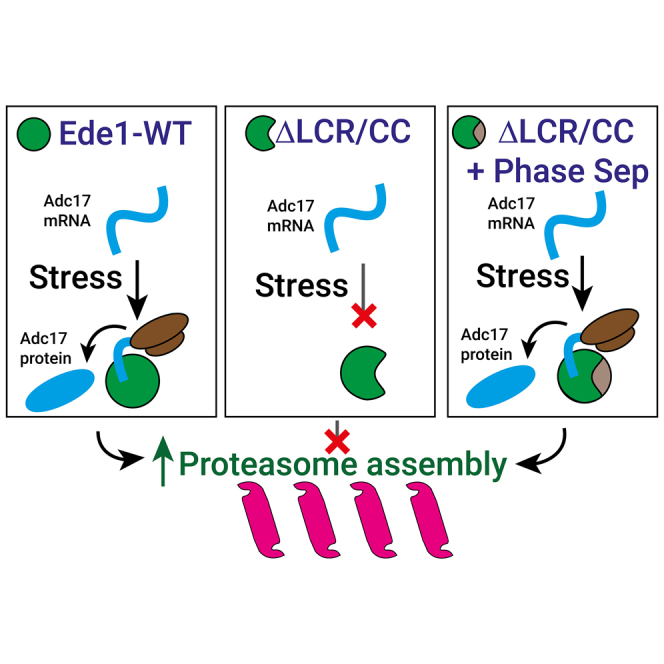

## Introduction

Cells exist in a state of constant flux, requiring turnover and adaptation of their protein content. Different proteins are required for progression through the various stages of the cell cycle, upon cellular stresses, and through development. Cells ensure they have the optimal amount of each protein under different conditions by a set of processes collectively termed proteostasis. Proteostasis impairment leads to a variety of disease states, particularly those associated with aging.^1^ The proteasome, composed of a core particle ‘capped’ by one or two regulatory particles, is a major contributor to proteostasis.^2^ Proteasomes are the major element responsible for degrading short-lived, misfolded, and orphan proteins to maintain the integrity and the stoichiometry of the proteome. Reduced proteasome load and activity can drive age-related diseases such as neurodegeneration,^3^ demonstrating the need to further understand mechanisms of proteasome regulation.

Upon conditions of cell stress, cells must rapidly adapt their proteome. They repress bulk protein synthesis, enhance stress-specific protein synthesis, and increase protein degradation. To efficiently increase protein degradation, cells temporarily enhance proteasome assembly.^2^^,^^4^^,^^5^ Proteasome assembly is a complex process requiring the action of several chaperones, which must be abundantly produced. Enhanced production of these assembly chaperones in yeast depends upon activation of the kinase Mpk1 (ERK5 in humans)^4^ and recruitment of the relevant mRNAs to densely packed actin structures termed cortical actin patches (CAPs) by the protein Ede1.^6^ Other mRNAs are recruited to similar actin-dense regions in both yeast and mammalian cells in unstressed conditions.^7^^,^^8^^,^^9^^,^^10^ How Ede1 mediates this process in the context of proteasome assembly chaperones (PACs) is unclear.

Ede1, and its mammalian homolog Eps15, is an early protein in clathrin mediated endocytosis (CME), involved in proper recruitment of several later-arriving proteins, endocytosis dynamics, and endocytosis initiation through the formation of phase separated punctae.^11^^,^^12^^,^^13^^,^^14^^,^^15^^,^^16^ The roles of Ede1 in endocytosis are mediated by different parts of the protein.^12^ Critically important are the central low complexity and coiled coil regions, which semi-redundantly allow formation of phase separated condensates to enhance endocytosis initiation and completion in both yeast and mammals.^11^^,^^15^ When defective CME occurs, Ede1 acts as an autophagy receptor for the resulting endocytic protein deposits (ENDs) through ATG8-interacting motifs in the C-terminal 50 amino acids.^17^ Deletion of either the low complexity or coiled-coil region diminishes END-like structure formation and affects localization to membrane puncta.^11^^,^^12^ Phase separation is driven by multiple weak interactions, and high local protein concentrations. Low complexity regions are particularly well implicated in driving phase separation, although they are neither necessary nor always sufficient for phase separation to occur.^18^ Other protein domains are also able to drive phase separation, notably oligomerization domains such as coiled coils.^19^ Intrinsically disordered phase separating regions have recently been implicated in RNA binding within structures that exhibit phase-separated properties, while RNA binding proteins are also enriched for coiled coil domains.^20^^,^^21^^,^^22^ We set out to determine how Ede1 controls PAC mRNA localization and translation, discovering a role for phase separation of the protein in regulating these processes.

## Results

### Low complexity and coiled coil regions of Ede1 are together necessary, but not sufficient, for rapamycin induced Adc17 expression

**Figure 1:**
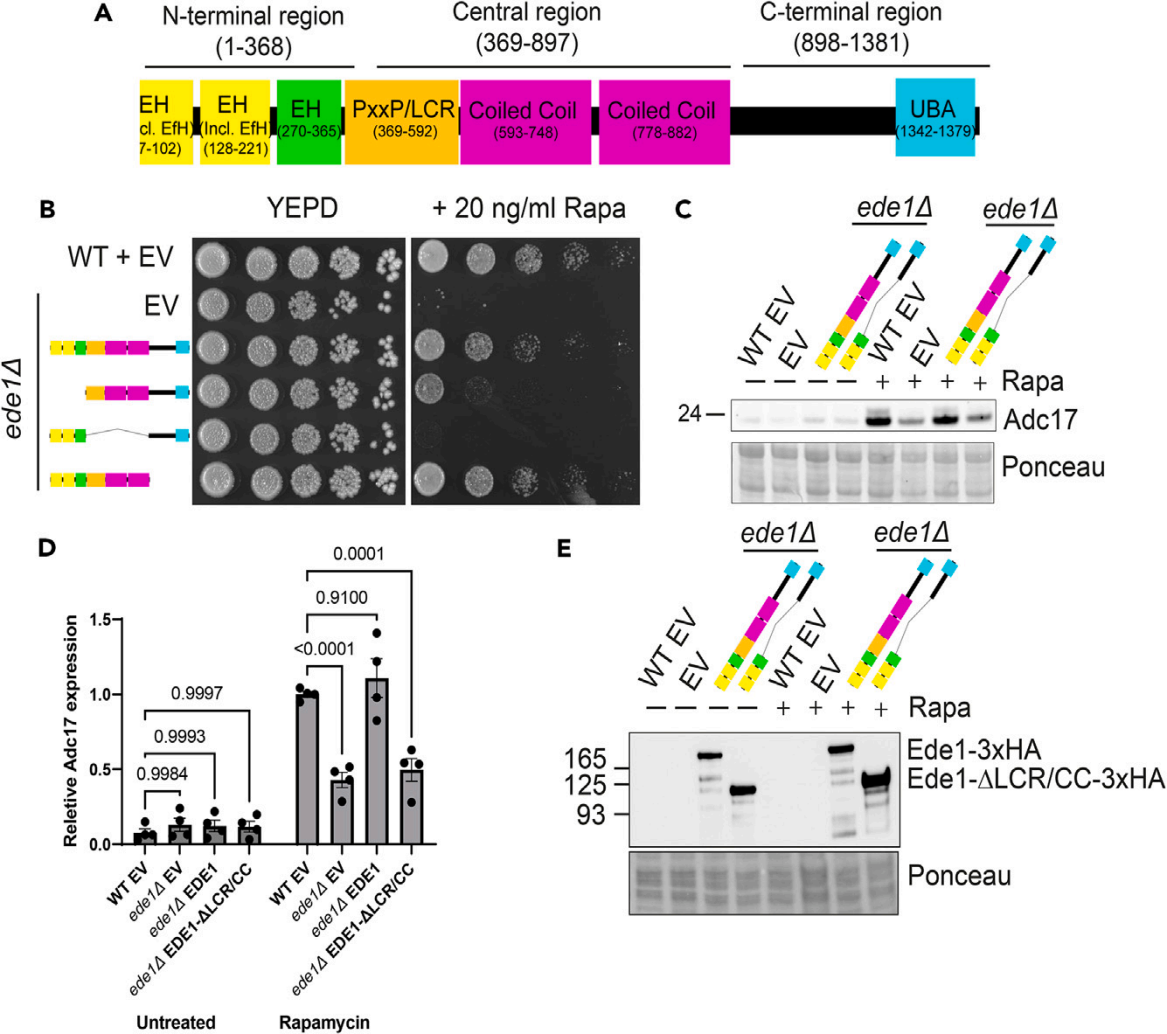
Ede1 central regions are necessary for Adc17 expression following rapamycin treatment (A) Domain architecture of Ede1. The N-terminal (EH), central (LCR/CC), and C-terminal regions which were deleted are highlighted. (B) Growth of WT + empty vector and *ede1Δ* cells rescued with Ede1 (empty vector, full length, ΔN-terminal EH domains, Δcentral low complexity region/coiled coil domains, and ΔC-terminal region) after 3 days on YEPD plates +/− rapamycin. (C) Adc17 expression levels in untreated and rapamycin WT + empty vector and treated *ede1Δ* cells rescued with empty vector, full length Ede1 and Ede1 lacking the central low complexity and coiled coil (LCR/CC) regions. (D) Quantification of C (n = 4), showing mean ± SEM. (E) Expression levels of Ede1-3xHA (full length and lacking the central low complexity region (LCR) and coiled coil (CC) domains) in untreated and rapamycin treated conditions.

To identify mechanisms underpinning Ede1-mediated recruitment of *ADC17* mRNA to cortical actin patches, we divided Ede1 into three parts (Figure 1A). As a first test, we examined the ability of constructs containing these truncated mutants to rescue growth of the *ede1Δ* mutant on plates containing the TORC1 inhibitor rapamycin (Figure 1B). Cells which lack the ability to increase proteasome assembly, including the *ede1Δ* strain, are expected to be unable to grow in these conditions.^4^^,^^6^ We found that the central low complexity/coiled coil (LCR/CC – amino acids 369–897) regions were, together, required to rescue *ede1Δ* cells. Constructs lacking the N-terminal EH domains (amino acids 2–368) had a partial defect in *ede1Δ* rescue, while the C-terminal region (amino acids 898–1381) was not required to rescue growth.

We examined Adc17 expression following rapamycin treatment when the truncated Ede1 proteins which were unable to fully rescue growth on rapamycin were expressed in *ede1Δ* cells. Ede1 lacking the N-terminal EH domains were expressed at comparable levels to full-length Ede1 and could rescue Adc17 expression (Figures S1A–S1C). In contrast, Ede1 lacking the LCR/CC regions was unable to rescue Adc17 expression (Figures 1C and 1D). The lack of rescue was not due to a lack of expression, as an HA tagged version was expressed at, if anything, higher levels than the full length (FL) version under both untreated and rapamycin treated conditions (Figure 1E), as previously reported.^11^ Surprisingly, constructs with individual deletions of either the LCR (amino acids 369–592) or CC (amino acids 593–897) were able to fully rescue growth and Adc17 induction of the *ede1Δ* mutant after rapamycin treatment (Figures S1D–S1F). Collectively, these data show that the LCR and CC regions of Ede1 are together necessary, but functionally redundant, for *ADC17* mRNA expression following rapamycin treatment.

### The phase-separation domains of Ede1 are not sufficient to induce Adc17 translation upon stress

**Figure 2:**
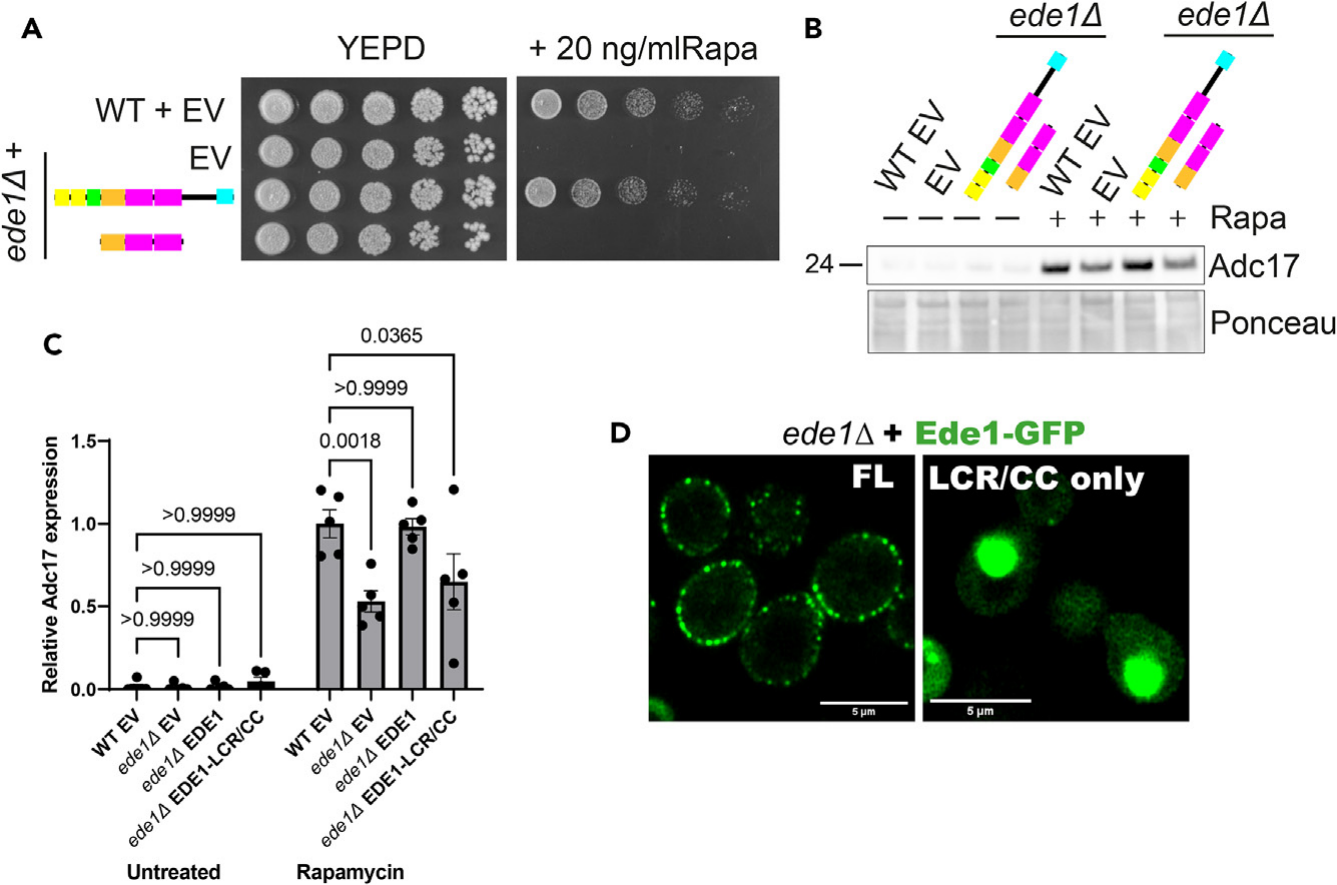
Ede1 central regions are not sufficient for Adc17 expression following rapamycin treatment (A) Growth of WT + empty vector and *ede1Δ* cells rescued with Ede1 (empty vector, full length, and LCR/CC alone) after 3 days on YEPD plates +/− rapamycin. (B) Adc17 expression levels in untreated and rapamycin treated WT + empty vector and *ede1Δ* cells rescued with empty vector, full length Ede1 and the Ede1 LCR/CC regions alone. (C) Quantification of B (n = 5), showing mean ± SEM. (D) Localization of Ede1-GFP (full length, LCR/CC alone) expressed from vectors in *ede1Δ* cells. Scale bars = 5 μm.

Having established the necessity of the LCR/CC domains for Ede1 function in Adc17 expression, we next tested their sufficiency by expressing this region under the control of the GPD promoter. This region of Ede1 alone was insufficient to rescue growth of *ede1Δ* cells on rapamycin containing plates (Figure 2A) or Adc17 expression after rapamycin treatment (Figures 2B and 2C). We then compared the localization of Ede1 full-length with that of LCR/CC domains alone. We found that full-length Ede1 is distributed in puncta at the cell periphery as reported previously,^11^^,^^12^ while the LCR/CC domains on their own form large cytoplasmic puncta (Figure 2D), most probably explaining why they are not sufficient for cell viability upon rapamycin treatment. Altogether, the LCR/CC domains are required, but functionally redundant, for Adc17 induction following rapamycin treatment. However, they are insufficient: requiring either the N- or C-terminus of Ede1.

### Disruption of phase separation prevents rapamycin induced Adc17 expression

We endeavored to find a common link between the LCR and CC domains of Ede1 which might explain their redundancy. Protein blast searches (NCBI) revealed no significant alignment. Reasoning that different sequences can have similar structural features, we assessed the predicted structures of each domain using ColabFold.^23^ As for the sequence identity, there were no obvious shared features between the two regions (Figures S1G and S1H).

**Figure 3:**
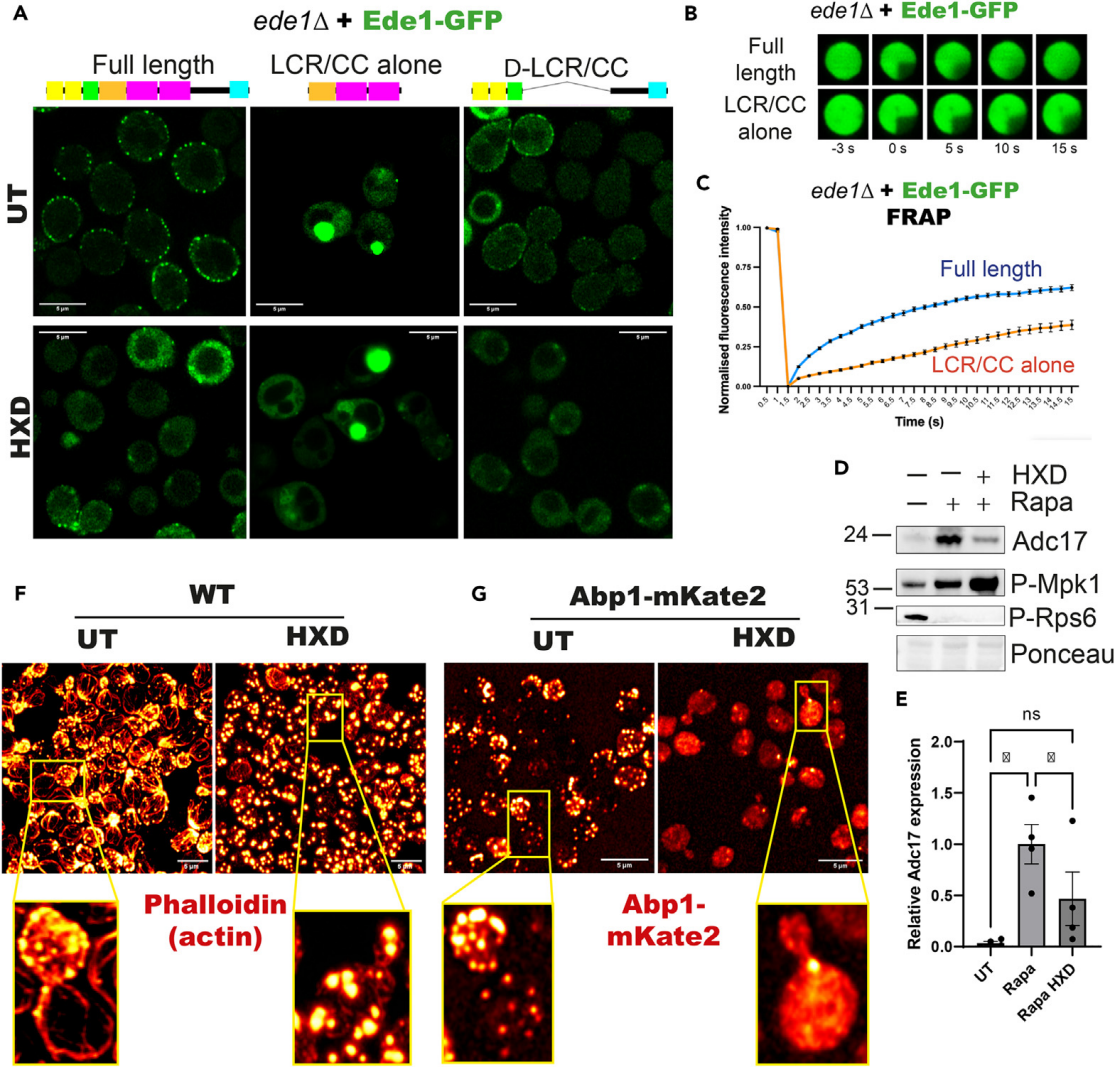
Disruption of phase separation prevents Adc17 expression and impacts Ede1 and actin structures (A) Effect of 1 h 5% 1,6-hexanediol (HXD) treatment on localization of Ede1-GFP (full length, ΔLCR/CC, LCR/CC alone) expressed from vectors in *ede1Δ* cells. Scale bars = 5 μm. (B) Representative images of FRAP experiments performed on punctae of Ede1-GFP (full length, LCR/CC alone). (C) Fluorescence Recovery After Photo bleaching graph for photobleached Ede1 (full length, LCR/CC alone) punctae. (D) Effect of treating WT cells with either rapamycin, or rapamycin and HXD on expression of Adc17, Mpk1 activation (measured by increasing levels of P-Mpk1), and TORC1 inhibition (measured by decreasing P-Rps6). (E) Quantification of Adc17 levels shown in B (n = 4), showing mean ± SEM. (F) Effect of 1 h 5% 1,6-hexanediol (HXD) treatment on the actin cytoskeleton, visualized by phalloidin staining, in WT cells. Scale bars = 5 μm. (G) Effect of 1 h 5% 1,6-hexanediol (HXD) treatment on the distribution of the endogenously tagged cortical actin patch-specific protein Abp1. Scale bars = 5 μm.

The LCR/CC regions of Ede1 have been implicated in phase separation of Ede1, allowing it to form structures at the endocytic patch.^11^ To assay whether this feature of Ede1 was required, we disrupted Ede1 phase separation pharmacologically. In untreated cells, Ede1 was distributed in puncta at the cell periphery as reported previously. Deletion of the LCR/CC regions caused Ede1 to localize largely to the cytosol, although we note punctate structures at the membrane which have not been observed before, likely due to the higher resolution afforded by Airyscan microscopy. When expressed on their own, the Ede1-LCR/CC regions form large cytoplasmic puncta (Figure 3A, top panels). Treatment of these cells with 5% 1,6-hexanediol (HXD) to disrupt phase separated structures caused the full length Ede1 to mainly disperse into the cytosol (Figures 2D and 3A, bottom panels), as previously observed.^11^ The small proportion of Ede1-ΔLCR/CC which formed into membrane puncta was similarly dispersed, while the Ede1-LCR/CC alone remained in large cytoplasmic puncta. To assess whether these structures were insoluble aggregates, we performed fluorescence recovery after photobleaching (FRAP) experiments. In agreement with our earlier results, Ede1 foci experienced fluorescence recovery, indicating they dynamically exchanged proteins with the surrounding milieu and are phase separated (Figures 3B and 3C). The foci produced by the LCR/CC truncation of Ede1 (half-time of 37.69 s) recovered less rapidly than full-length Ede1 (half-time of 4.55 s), as expected from our previous results. The half-life of full-length Ede1 is consistent with previous studies reporting a half-life in the 2–8 s range.^11^^,^^17^ However, we still observed recovery for the LCR/CC truncation of Ede1, indicating that these structures are not insoluble aggregates. We predicted that the observed disruption of Ede1 structures would in turn disrupt the induction of Adc17 expression after rapamycin treatment. We therefore co-treated WT cells with rapamycin and HXD. As expected, this prevented the rapamycin-induced increase in Adc17 expression, while maintaining the upstream signaling elements (Mpk1 activation and TORC1 inhibition) required for Adc17 induction (Figures 3D and 3E).^4^ We examined the effect of HXD on actin cytoskeletal structures and found cellular actin was predominantly in large aggregates (Figure 3F), with few clear cortical actin patches or actin cables. These data indicate that Adc17 expression is not induced purely by mRNA removal from actin cables, which we had previously been unable to rule out.^6^

As the large aggregates seen could be cortical actin patches, we assessed the localization of the actin patch specific marker Abp1 (Figure 3G). Instead of being confined to the small punctate actin patch structures, Abp1 was almost exclusively cytosolic after HXD treatment, although there remained a limited number of actin patch-like structures. Overall, disrupting phase separation disrupts cell actin architecture, with the near absence of cortical actin patches necessarily preventing *ADC17* mRNA recruitment to these structures to initiate translation. Similar observations have been made in mammalian systems, where actin nucleators can rely on phase separation for function.^24^^,^^25^ This is consistent with a role for Ede1 phase separation in mediating Adc17 expression, although as HXD will indiscriminately affect many other cellular processes we cannot exclude additional perturbations affecting Adc17 expression.

### Improving phase separation of the Ede1-ΔLCR/CC mutant rescues Adc17 expression

**Figure 4:**
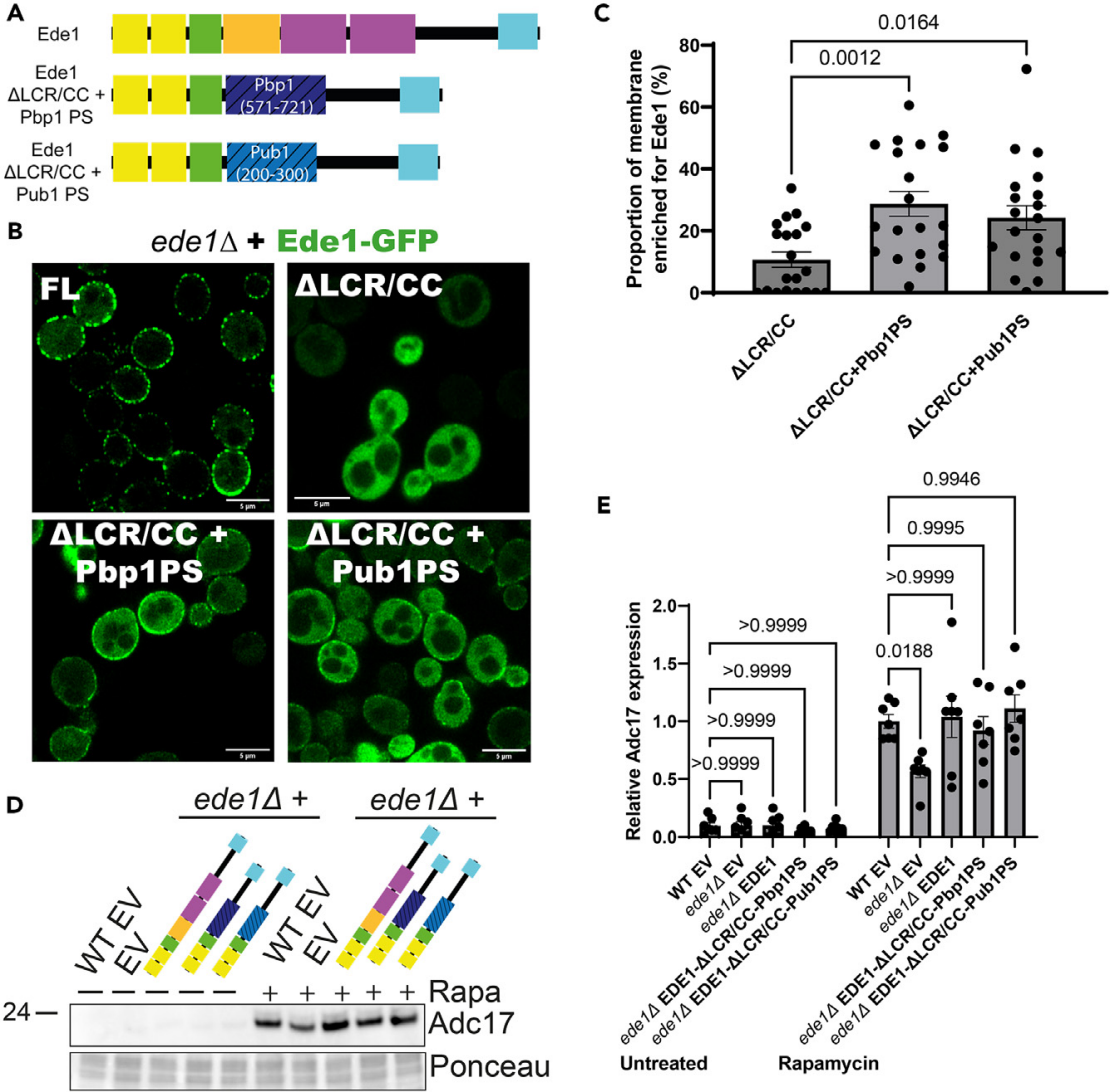
Restoring Ede1 phase separation restores Adc17 induction upon rapamycin treatment (A) Domain architecture of Ede1 full length compared with constructs where the LCR/CC regions have been replaced with phase separating regions of Pbp1 and Pub1. (B) Localization of Ede1-GFP (full length, ΔLCR/CC, ΔLCR/CC + Pbp1 PS, and ΔLCR/CC + Pub1 PS) in *ede1Δ* cells. Scale bars = 5 μm. (C) Proportion of membrane enriched >1.5-fold over the cytoplasm for Ede1-mutant-GFP. Mean ± SEM is shown, n = 20 cells – 5 each from 4 independent replicates. (D) Adc17 expression levels in untreated and rapamycin treated WT + empty vector and *ede1Δ* cells rescued with empty vector, full length Ede1 and Ede1 where the LCR/CC domains have been replaced with the phase separating regions of Pbp1 and Pub1. (E) Quantification of C (n = 7), showing mean ± SEM.

As our data indicate that phase separation of Ede1 is important for Adc17 expression after rapamycin treatment, we questioned whether we could rescue the functionality of the Ede1 lacking the LCR/CC regions by incorporating phase separation domains from other proteins (Figure 4A). This will allow us to differentiate the effect of altering the phase separating properties of Ede1, and other aspects likely mediated by these regions including specific protein-protein interactions. We chose phase separating regions from the RNA binding proteins Pbp1 and Pub1, as these have been defined.^26^^,^^27^ The Pbp1 phase separating region (Pbp1-PS, amino acids 571–721) is a low complexity sequence containing no known RNA binding motifs, while the Pub1-PS (amino acids 200–300) has part of an RNA binding motif and a low complexity sequence. Importantly, mutant analysis indicated that neither Pub1 nor Pbp1 are involved in regulating Adc17 expression upon rapamycin treatment (Figures S2A–S2C). Both phase separating regions are intrinsically disordered, containing no coiled coil structures. The low-complexity part of the Ede1 central region additionally contains 3 PxxP motifs, which may be important for protein-protein interactions. Pbp1-PS contains 4 such motifs, while Pub1-PS contains 0; allowing us to determine whether these motifs are important for Adc17 expression (Figure S2D). When performing sequence alignments using the NCBI blast tool, we observed that sequence conservation was extremely low to non-existent (Figures S2E and S2F). While these regions share the ability to form phase separated structures, the distinct sequence identities will likely fail to restore any specific interactions barring PxxP motif-mediated interactions in constructs containing Pbp1-PS, but not Pub1-PS.

Introducing both Pbp1-PS and Pub1-PS partially rescued Ede1-ΔLCR/CC localization (Figures 4B and 4C). Notably, neither was enough to restore localization completely. A similar pattern of localization rescue was observed when introducing prion-like domains to Ede1-ΔLCR/CC.^11^ We next assessed whether Ede1 containing these different phase separation domains could rescue Adc17 expression following rapamycin treatment in *ede1Δ* cells. We found that Adc17 expression was significantly restored in both cases (Figures 4D and 4E). This indicates that an important role of the LCR/CC regions in the context of mediating Adc17 expression is through their ability to drive phase separation of Ede1. Interestingly, expression of these chimeric Ede1 proteins partly restored growth of *ede1Δ* cells upon rapamycin-containing plates (Figure S2G), indicating that partly restoring Ede1 phase-separation at the membrane is beneficial for rapamycin survival. The fact that growth rescue was incomplete suggests that additional functionality for the LCR/CC regions of Ede1 in rapamycin resistance is needed. This added requirement for growth could be through specific protein-protein interactions which are not restored by increasing phase separation propensity in this manner.

### Ede1 phase separation is important for mRNA recruitment to translational hotspots

Having defined the important elements and properties of Ede1 for *ADC17* mRNA translation following rapamycin treatment, we wanted to establish why these aspects were important. As we have previously shown that full length Ede1 dynamically interacts with *ADC17* mRNA and is required to recruit *ADC17* mRNA to cortical actin patches following stress, it seems likely that this interaction and subsequent recruitment will be perturbed without the LCR/CC domains.

**Figure 5:**
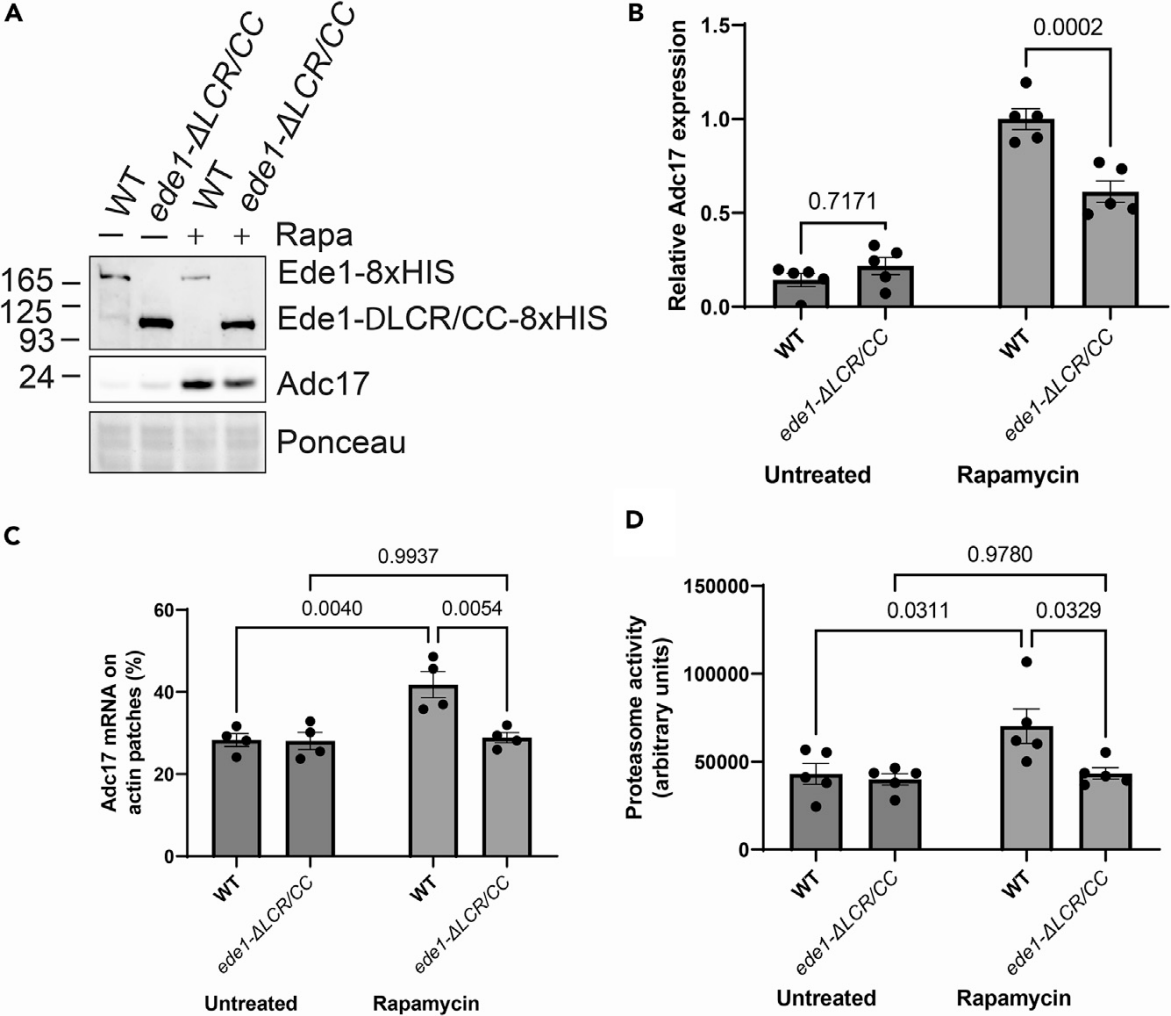
Ede1 LCR/CC domains mediate ADC17 mRNA recruitment to cortical actin patches and are important for effective enhancement of proteasome activity on stress (A) Expression of Adc17 and Ede1-8xHis (full length and ΔLCR/CC) in untreated and rapamycin treated cells. (B) Quantification of A (n = 5), showing mean ± SEM. (C) Proportion of *ADC17* mRNA localized at cortical actin patches in untreated and 1 h rapamycin treated cells with WT and ΔLCR/CC Ede1 (n = 4), showing mean ± SEM. (D) Proteasome activity of WT and *ede1-ΔLCR/CC* cells under untreated and rapamycin treated conditions (n = 5), showing mean ± SEM.

To test this hypothesis, we used CRISPR/Cas9^28^ to first add an 8xHIS tag to endogenous Ede1, allowing its detection by Western blot. Ede1-8xHIS was expressed, and cells containing it both could induce Adc17 expression and were able to grow on rapamycin containing plates, together demonstrating that it is functional (Figures 5A, 5B, and S3A). To confirm that endogenous deletion of Ede1 phase separation domain is abrogating Ede1 function the LCR/CC domains were then deleted using the same methodology. These tagged proteins were expressed, with elevated expression of the ΔLCR/CC mutant (Figure 5A). The ΔLCR/CC strain was unable to grow on rapamycin plates (Figure S3A) or induce Adc17 expression following rapamycin treatment (Figures 5A and 5B). This confirms that the phase separation domain of Ede1 is physiologically important for its function upon stress.

We next tested whether strains containing these mutant Ede1 proteins were capable of recruiting *ADC17* mRNA to cortical actin patches following rapamycin treatment. We deleted the endogenous Ede1 LCR/CC domains in cells containing an endogenous knock-in of PP7 tags on the 3′UTR of *ADC17*, allowing mRNA labeling by overexpression of PCP-GFP.^6^ Consistent with the lack of Adc17 expression observed, these mutants failed to recruit *ADC17* mRNA to cortical actin patches (Figure 5C), as observed for *ede1*Δ cells.^6^ The *ADC17* mRNA remained highly associated with actin cables, with a negligible proportion not associated with any actin structures (Figures S3B and S3C), indicating there was no issue with the ability of the mRNA to interact with actin. Rather, without the LCR/CC domains, Ede1 is unable to mediate stress-induced *ADC17* mRNA recruitment to cortical actin patches, as previously reported for *ede1Δ* cells.^6^

Finally, to understand the biological relevance of these observations, we analyzed the stress induced activation of proteasome assembly, which is dependent upon the induction of assembly chaperones.^2^ As previously reported, in WT cells there is a significant rise in the levels of proteasome activity following rapamycin treatment (Figure 5D). This induction was compromised in the *ede1-ΔLCR/CC* mutant. Altogether, this work demonstrates the requirement of Ede1 phase separation for proteasome homeostasis following rapamycin treatment via recruitment of assembly chaperone mRNAs to cortical actin patches for translation.

## Discussion

We have here demonstrated a critical function of phase separation underpinning *S. cerevisiae*’s ability to recruit mRNA to cortical actin patches for translation under stress conditions. mRNAs being recruited to phase separating membrane-less structures have been extensively described, most notably in the cases of stress granules (SGs) and P-bodies (PBs).^29^ In contrast to the work described here, these structures are correlated with translation downregulation and mRNA degradation respectively, although not all mRNAs associated with these structures experience reduced translation.^29^^,^^30^^,^^31^ Translation within phase separated structures has been described for some mRNAs, aiding the formation of certain protein complexes.^20^ It is conceivable that a similar scenario may help in assembling proteasome regulatory particles, although it should also be noted that the interaction between *ADC17* mRNA and Ede1 protein is highly dynamic.^6^

This work has demonstrated that *ADC17* mRNA recruitment to cortical actin patches does not rely on a particular protein sequence, but rather the ability of Ede1 to phase separate (Video S1). In agreement with previous work, we found that the single deletion of the LCR or CC domain is not sufficient to prevent Ede1 phase separation and PAC induction. This suggests that each domain may promote phase separation by mediating many small interactions, as widely reported for this process. In this scenario, it is only the absence of both domains which disrupts Ede1 oligomerization and its subsequent phase separation. This agrees with recent work identifying intrinsically disordered regions as important for the non-specific interaction of unconventional RNA binding proteins with mRNA.^32^ While protein phase separation regulates mRNA recruitment to PBs and SGs upon stress, it is not apparent how *ADC17* mRNA interacts more with Ede1 to boost its recruitment to cortical actin patches upon stress. It is possible that actin cable disruption following stress triggers *ADC17* mRNA recruitment to cortical actin patches and that Ede1 is stabilizing it at these structures for translation initiation. Ede1 localization is similar between untreated and rapamycin-treated conditions,^6^ indicating likely additional regulation we have yet to identify. This additional regulation could be through post-translational modifications of Ede1,^33^^,^^34^^,^^35^ changes in interacting proteins, altered endocytosis,^36^ or downregulation of other mRNAs which may have a stronger interaction with Ede1 under optimal conditions.^37^^,^^38^

Video S1. Summary video file describing how Ede1 controls ADC17 mRNA translation upon stress

Recruitment to actin-rich tight junctions for translation has been described for the ZO-1 mRNA in mammalian cells. In a striking parallel with what we have observed, this recruitment is mediated by the phase separating mRNA binding protein CPEB1.^7^^,^^39^ Furthermore, proteins associated with actin-rich structures which contain disordered regions such as Zyxin, SNTB2, and Ezrin have been found to bind RNA.^40^^,^^41^^,^^42^ Zyxin has further been shown to be capable of phase separating with the scaffolding protein LIMD1, which is recruited to focal adhesions depending on cell substrate stiffness.^43^

This work identifies protein phase separation-mediated mRNA recruitment as important in stress-regulated translation at actin structures. When combined with other work, this suggests phase-separated mediated recruitment of mRNAs to actin-rich structures for translation may be widespread across life. Further work remains to identify just how many mRNAs are regulated by phase separation recruitment to actin-rich structures but, as Ede1 is highly enriched in the RNA-bound proteome,^37^ this is likely to regulate many more processes.

### Limitations of the study

Although this work discovers that Ede1 phase separation is essential for the regulation of proteasome assembly and activity upon rapamycin treatment, the molecular attributes responsible for Ede1 phase separation could not be elucidated. This was mainly due to the lack of structural motifs present in the LCR/CC region. Better understanding how low complexity domains drive phase separation of proteins will be important in the future. Whether Ede1-mRNA interaction contributes to phase separation and what are the elements on ADC17 mRNA important for translation regulation are also important queries to solve in the future.

## STAR★Methods

### Key resources table

**Table:** 

REAGENT or RESOURCE	SOURCE	IDENTIFIER
**Antibodies**		

Anti Adc17 (1:200, Sheep)	DSTT	DU66321
Anti HA (1:1000, Mouse)	Santa Cruz	Sc-7392
Anti P-Mpk1 (1:1000, Rabbit)	CST	4370S
Anti P-Rps6 (1:1000, Rabbit)	CST	2211
Anti His (1:2000, Mouse)	ThermoFisher	MA1-21315
HRP conjugated anti Mouse (1:10,000)	CST	7076S
HRP conjugated anti Rabbit (1:10,000)	CST	7074S
HRP conjugated anti Sheep (1:5,000)	Sigma-Aldrich	A3415

**Chemicals, peptides, and recombinant proteins**		

Rapamycin	LC Laboratories	R-5000
Concanavalin A	Sigma-Aldrich	C2010
1,6-hexanediol	Sigma-Aldrich	H11807
Suc-LLVY-AMC fluorogenic substrate	Cambridge Biosciences	CAY37043

**Experimental models: Organisms/strains**		

BY4741 (WT)	Horizon Discovery	N/A
BY4741 *ede1Δ::KanMX*	Horizon Discovery	N/A
BY4741 *abp1-mKate2::KanMX*	This work	N/A
BY4741 *pbp1Δ::KanMX*	Horizon Discovery	N/A
BY4741 *pub1Δ::KanMX*	Horizon Discovery	N/A
BY4741 *ede1-8xHIS*	This work	N/A
BY4741 *ede1-ΔLCR/CC-8xHIS*	This work	N/A
BY4741 *adc17-24xSL::KanMX*	[5]	N/A
BY4741 *adc17-24xSL::KanMX ede1-ΔLCR/CC*	This work	N/A

**Oligonucleotides**		

See Table S1 for oligonucleotide sequences.		

**Recombinant DNA**		

p416 (EV)	[4]	N/A
p416-Ede1	[5]	N/A
p416-Ede1-ΔEH	This study	N/A
p416-Ede1-ΔLCR/CC	This study	N/A
p416-Ede1-ΔCTD	This study	N/A
p416-Ede1-ΔLCR/CC-3xHA	This study	N/A
p416-Ede1-3xHA	This study	N/A
p416-Ede1-ΔEH-3xHA	This study	N/A
p416-Ede1-ΔLCR	This study	N/A
p416-Ede1-ΔCC	This study	N/A
p416-Ede1-LCR/CC	This study	N/A
p416-Ede1-GFP	This study	N/A
p416-Ede1-ΔLCR/CC-GFP	This study	N/A
p416-Ede1-LCR/CC-GFP	This study	N/A
p416-Ede1-ΔLCR/CC-Pbp1PS	This study	N/A
p416-Ede1-ΔLCR/CC-Pub1PS	This study	N/A
p416-Ede1-ΔLCR/CC-Pbp1PS-GFP	This study	N/A
p416-Ede1-ΔLCR/CC-Pub1PS-GFP	This study	N/A
pFA6-3xHA-mKate2-KanMX	This study	N/A
pML104-Ede1-8xHIS-gRNA	This study	N/A
pML104-Ede1-ΔLCR/CC-gRNA	This study	N/A
pFA6-Cyc1pr-PP7-2xeGFP	[5]	N/A

**Deposited data**		

Unprocessed images	This study	https://doi.org/10.17632/77hfnp3fxm.1

**Software and algorithms**		

Zen	Zeiss	2.3 SP1 FP3
FIJI	ImageJ	Win64
Prism	GraphPad	Version 9
Blastp	NCBI	https://blast.ncbi.nlm.nih.gov/
Snapgene	GSL Biotech	5.3.2

### Resource availability

#### Lead contact

Further information and requests for resources should be directed to the lead contact, Adrien Rousseau (arousseau@dundee.ac.uk).

#### Materials availability

Materials established in this paper will be shared by the lead contact upon request.

#### Data and code availability

• All data reported in this paper will be shared by the lead contact upon request.
• This paper does not report any original code.
• Unprocessed images used in this study can be accessed at Mendeley Data (https://doi.org/10.17632/77hfnp3fxm.1).

### Experimental model and subject details

All *Saccharomyces cerevisiae* strains were obtained or derived from those in the BY4741 Mata knock-out mutant collection. Growth was performed at 30°C either on YPD agar plates or YEPD liquid culture, with 200 rpm shaking. For microscopy, cells were grown overnight in SC medium and readjusted to exponential phase before imaging. Where drop assays were performed, spots were diluted 1:5 from an initial OD_600 nm_ of 0.2 and incubated at 30°C for 3 days unless otherwise indicated before imaging.

### Method details

#### Cloning

All cloning was performed using the Infusion system (Takara, 638909) and Stellar competent cells (Takara, 636763) as described by the manufacturer. PCR inserts were generated using GXL polymerase (Takara, R051A) or KOD HotStart (Millipore, 71086) and extracted using a gel extraction kit (Zymogen, D4002). Minipreps were performed using the Monarch system (NEB, T1010L) with vector backbones generated by EcoRI restriction enzyme digestion (ThermoFisher, FD0274, as per manufacturer’s instructions) or PCR with GXL polymerase/HiFi polymerase (Takara, R051A/639298). For the Pbp1-PS/Pub1-PS insertions into Ede1, a BshTI site was generated within the Ede1 construct. The PS domains were inserted into BshTI (ThermoFisher, FD1464) digested vector, removing the remnants of the restriction site. All obtained vectors were sequenced to confirm accuracy.

#### Mutant generation

mKate2 tagging of Abp1 was performed by homologous recombination as described.^6^ Ede1-8xHis tagged mutants were generated using CRISPR/Cas9 as described^28^ and accuracy confirmed using DNA sequencing and Western blot. A second CRISPR/Cas9 deletion was performed as before to delete the low complexity and coiled coil regions, which was again validated with DNA sequencing and Western blot.

#### Cell treatment for protein analysis

Cells were adjusted to 0.2 OD_600 nm_ from overnight growth either on YEPD agar plates or YPD medium and placed shaking 200 rpm 30°C. When they were growing logarithmically (typically after ∼4 h), they were diluted once again to 0.2 OD_600 nm_ and split into 12 ml (treated with 0.2 μM Rapamycin) and 4 ml (left untreated) before harvesting after 4 h. Harvesting was performed by spinning down at 3,200*g* for 3–4 min at 4°C, then the pellets flash-frozen in dry ice. When 1,6-hexanediol was used in conjunction with the rapamycin, this was added to 5% from either a 20 or 50% stock solution in YPD at the same time as rapamycin treatment.

#### Protein extraction

Sample pellets were defrosted on ice, then washed in 600 μl ice-cold water (6,200*g*, 30 sec) and supernatant removed. Pellets were resuspended in 400 μl ice-cold 2 M LiAc, spun down again and the supernatant removed. Pellets were resuspended in 400 μl ice-cold 0.4 M NaOH, spun down again and the supernatant removed. Pellets were then resuspended in 110 μl lysis buffer (0.1 M NaOH, 0.05 M EDTA, 2% SDS, 2% β-mercaptoethanol, PhosStop (Roche) and cOmplete protease inhibitor cocktail (Roche)) and boiled for 10 min. 2.64 μl 4 M acetic acid was added, the samples vortexed then boiled for a further 10 min. Samples were spun down (17,000*g*, 5 min) and 80 μl mixed with 20 μl 5x sample loading buffer (0.25 M Tris–HCl pH 6.8, 50% glycerol, 0.05% bromophenol blue). Concentration was measured from the remaining sample using a nanodrop (Thermofisher) *A*_280 nm_. All samples were adjusted to the same concentration and stored at −20°C.

#### Western blotting & protein visualisation

Samples (∼30 μg protein in each) were run on home-made 6–14% or 6–20% Bis-Tris acrylamide gels, poured as described^6^ at 120V for 1.5–2 hours in MES-SDS running buffer (Formedium). Gels were incubated in transfer buffer (1x Nupage transfer buffer, NP0006-1, 20% Ethanol) and semi-dry blotting performed using blotting paper (Invitrogen, LC2008) and 0.2 μm nitrocellulose (BioRad 1620112) which had been pre-soaked in transfer buffer at 25V for 15 or 30 min on the BioRad TurboBlot system. Membranes were stained with ponceau S solution (Santa Cruz, sc-301558), imaged, then soaked in TBS-T (TBS +0.1% Tween 20) containing 5% milk powder for at least 1 hour, before washing and incubating with relevant primary antibodies (see key resources table) overnight. Antibodies were then removed, membranes washed, and incubated with secondary antibodies (see key resources table) for at least an hour before imaging. All membrane imaging was performed on a Chemidoc Touch system (BioRad) using Clarity ECL (BioRad).

#### Live cell microscopy

Cells were grown overnight in SC medium, diluted down to ∼0.2 OD_600 nm_, then grown for 3–4 h. Cells were then attached to a 35 mm fluorodish (Fisher Scientific) which had been pre-incubated with Concanavalin-A (500 μl, 1 mg/ml, Sigma Aldrich C2010), excess of which was removed by washing. Cells were allowed to attach for 0.5–1 hour, then washed twice with pre-warmed SC. Cells were then left untreated or treated for 1 h with 5% 1,6-hexanediol and imaged on a Zeiss 880 Airyscan microscope (Airyscan mode, Alpha Plan-APO 63×/1.4 oil objective (Zeiss)) with Zen 2.3 SP1 FP3 software at 30°C, through the mid-section of the cells. Subsequent image processing was performed using FIJI.

#### Fluorescence recovery after photo bleaching (FRAP)

FRAP experiments were performed on a Zeiss 880 Airyscan microscope (Airyscan mode, Alpha Plan-APO 100×/1.46 oil DIC VIS objective (Zeiss) and Alpha Plan-APO 63×/1.4 oil objective (Zeiss)). For bleaching the laser power was set to maximum. Three images were taken before the bleaching and 27 images after bleaching at time intervals of 0.5 s at 1% laser transmission to avoid additional bleaching. Fluorescence recovery analyses of the bleached areas were carried out using ImageJ Stowers plugins (Stowers institute for medical research). The averaged recovery curves within the bleached region were fit to a single rising exponential. The half-life (t1/2) value was defined as the time required for reaching half-maximum recovery and was calculated from the corrected recovery curve.*Fixed cell microscopy*.

Cells were grown overnight in YEPD medium, or on YPD plates then adjusted to 0.2 OD_600 nm_ in YEPD medium and placed at 30°C, 200 rpm until they reached logarithmic growth. 4 ml cells were either treated with 0.2 μM rapamycin or 5% 1,6-hexanediol for 1 hour before being fixed or fixed immediately with formaldehyde (final concentration 3.7%) for 20 mins at 30^o^C 200 rpm. Cells were pelleted in a benchtop centrifuge (7,800g, 2 min), the supernatant was discarded then the pellets washed twice with 6 ml PBS. Where actin staining was used, pellets were then resuspended in a 1.5 ml Eppendorf in 100 μl PBS + 0.1% Triton X-100 + 1:1000 Rhodamine Phalloidin (Abcam; ab255138) and incubated on a rotating shaker in the dark for 1 h. For mounting onto slides, cells were spun down (7,800*g*, 3 min), washed once with 1 ml PBS, and resuspended in 6 μl ProLong Glass antifade mounting medium (Thermo Fisher, P36980), pipetted onto a SuperFrost microscope slide (VWR, 631-0847) and covered with a glass coverslip (VWR, 631-0119). Samples were stored in the dark at room temperature overnight, then moved to 4°C in the dark. Z-stacks of cells were taken using a Zeiss 880 Airyscan microscope (Airyscan mode, Alpha Plan-APO 63×/1.4 oil objective (Zeiss)) with Zen 2.3 SP1 FP3 software at room temperature. Images were analysed as described.^6^

#### Proteasome activity assays

Cells were grown overnight in YEPD liquid medium (200 rpm, 30°C), diluted to 0.2 OD_600 nm_ and placed back growing at 32°C, 200 rpm for 3-4 hours to allow them to reach logarithmic growth (∼0.5–0.6 OD_600 nm_). Cells were then diluted to 0.3 OD_600 nm_ and either treated with 0.2 μM Rapamycin (30 ml) or left untreated (15 ml) and returned to the incubator. After 3 hours cells were spun down (4°C, 3200*g*, 3 min), resuspended in 800 μl ice-cold water, transferred to a 2 ml tube and spun down again (4°C, 6200*g*, 30 sec). Cells were then resuspended in 300 μl native lysis buffer (50 mM Tris pH 8, 5 mM MgCl_2_, 0.5 mM EDTA, 5% glycerol, 1 mM DTT and 5 mM ATP) and transferred to a 2 ml tube containing 250 μl acid washed glass beads (Sigma-Aldrich, G-8772). Cells were then lysed by bead beating (3 × 30 sec on, 5 min off in FastPrep 24, 4.0 M/S at 4°C), and the lysates spun down (4°C, 17,000*g*, 2 min), transferred to a fresh tube and spun down again (4°C, 17,000*g*, 10 min). Protein concentrations were assessed using a nanodrop *A*_280_ (ThermoFisher), and ∼50 mg was added in triplicate to a black bottom 96-well plate. 100 μl pre-warmed assay buffer (50 mM Tris–HCl pH 7.5, 150 mM NaCl, 5 mM MgCl_2_, 10% glycerol containing 0.1 mM suc-LLVY-AMC fluorogenic substrate (Cambridge Biosciences, CAY37043) was added and chymotrypsin activity measured every 5 minutes for 45 minutes using a Fluostar Omega microplate reader (BMG Labtech).

### Quantification and statistical analysis

Western blot quantification was performed using the FIJI measure tool. Average band intensity was measured, the background subtracted and then normalised for the loading measured similarly using the Ponceau stain.

The proportion of Ede1 enriched at the membrane was calculated using FIJI. The signal intensity of each pixel at the cell membrane was taken and divided by a cytosolic signal average. The proportion of membrane pixels which showed an enrichment of >1.5fold compared to the cytosol was then calculated.

All statistical analysis was carried out using Graphpad prism, with one- or two-way anova as appropriate, followed by multiple comparison t-test analysis. Where individual experiments are shown (e.g. drop assays), they are representative of at least 3 independent replicates.

## References

[1] Kaushik S., Cuervo A.M. (2015). Proteostasis and aging. Nat. Med..

[2] Rousseau A., Bertolotti A. (2018). Regulation of proteasome assembly and activity in health and disease. Nat. Rev. Mol. Cell Biol..

[3] Tanaka K., Matsuda N. (2014). Proteostasis and neurodegeneration: The roles of proteasomal degradation and autophagy. Biochim. Biophys. Acta.

[4] Rousseau A., Bertolotti A. (2016). An evolutionarily conserved pathway controls proteasome homeostasis. Nature.

[5] Waite K.A., Burris A., Vontz G., Lang A., Roelofs J. (2022). Proteaphagy is specifically regulated and requires factors dispensable for general autophagy. J. Biol. Chem..

[6] Williams T.D., Cacioppo R., Agrotis A., Black A., Zhou H., Rousseau A. (2022). Actin remodelling controls proteasome homeostasis upon stress. Nat. Cell Biol..

[7] Nagaoka K., Udagawa T., Richter J.D. (2012). CPEB-mediated ZO-1 mRNA localization is required for epithelial tight-junction assembly and cell polarity. Nat. Commun..

[8] Katz Z.B., Wells A.L., Park H.Y., Wu B., Shenoy S.M., Singer R.H. (2012). β-Actin mRNA compartmentalization enhances focal adhesion stability and directs cell migration. Genes Dev..

[9] Williams T.D., Rousseau A. (2022). Actin dynamics in protein homeostasis. Biosci. Rep..

[10] Boraas L., Hu M., Thornton L., Vejnar C.E., Zhen G., Giraldez A.J., Mayr C., Wang S., Nicoli S. (2021). Non-coding function for mRNAs in Focal Adhesion Architecture and Mechanotransduction. bioRxiv.

[11] Kozak M., Kaksonen M. (2022). Condensation of Ede1 promotes the initiation of endocytosis. Elife.

[12] Lu R., Drubin D.G. (2017). Selection and stabilization of endocytic sites by Ede1, a yeast functional homologue of human Eps15. Mol. Biol. Cell.

[13] Stimpson H.E.M., Toret C.P., Cheng A.T., Pauly B.S., Drubin D.G. (2009). Early-arriving Syp1p and Ede1p function in endocytic site placement and formation in budding yeast. Mol. Biol. Cell.

[14] Carroll S.Y., Stimpson H.E.M., Weinberg J., Toret C.P., Sun Y., Drubin D.G. (2012). Analysis of yeast endocytic site formation and maturation through a regulatory transition point. Mol. Biol. Cell.

[15] Day K.J., Kago G., Wang L., Richter J.B., Hayden C.C., Lafer E.M., Stachowiak J.C. (2021). Liquid-like protein interactions catalyse assembly of endocytic vesicles. Nat. Cell Biol..

[16] Kaksonen M., Roux A. (2018). Mechanisms of clathrin-mediated endocytosis. Nat. Rev. Mol. Cell Biol..

[17] Wilfling F., Lee C.W., Erdmann P.S., Zheng Y., Sherpa D., Jentsch S., Pfander B., Schulman B.A., Baumeister W. (2020). A Selective Autophagy Pathway for Phase-Separated Endocytic Protein Deposits. Mol. Cell.

[18] Zhang H., Ji X., Li P., Liu C., Lou J., Wang Z., Wen W., Xiao Y., Zhang M., Zhu X. (2020). Liquid-liquid phase separation in biology: mechanisms, physiological functions and human diseases. Sci. China Life Sci..

[19] Zeng M., Shang Y., Araki Y., Guo T., Huganir R.L., Zhang M. (2016). Phase Transition in Postsynaptic Densities Underlies Formation of Synaptic Complexes and Synaptic Plasticity. Cell.

[20] Luo Y., Pratihar S., Horste E.H., Mitschka S., Mey A.S.J.S., Al-Hashimi H.M., Mayr C. (2023). mRNA interactions with disordered regions control protein activity. bioRxiv.

[21] Ma W., Mayr C. (2018). A Membraneless Organelle Associated with the Endoplasmic Reticulum Enables 3'UTR-Mediated Protein-Protein Interactions. Cell.

[22] Ford L.K., Fioriti L. (2020). Coiled-Coil Motifs of RNA-Binding Proteins: Dynamicity in RNA Regulation. Front. Cell Dev. Biol..

[23] Mirdita M., Schütze K., Moriwaki Y., Heo L., Ovchinnikov S., Steinegger M. (2022). ColabFold: making protein folding accessible to all. Nat. Methods.

[24] Yang S., Liu C., Guo Y., Li G., Li D., Yan X., Zhu X. (2022). Self-construction of actin networks through phase separation–induced abLIM1 condensates. Proc. Natl. Acad. Sci. USA.

[25] Case L.B., Zhang X., Ditlev J.A., Rosen M.K. (2019). Stoichiometry controls activity of phase-separated clusters of actin signaling proteins. Science (New York, N.Y.).

[26] Yang Y.S., Kato M., Wu X., Litsios A., Sutter B.M., Wang Y., Hsu C.H., Wood N.E., Lemoff A., Mirzaei H. (2019). Yeast Ataxin-2 Forms an Intracellular Condensate Required for the Inhibition of TORC1 Signaling during Respiratory Growth. Cell.

[27] Gotor N.L., Armaos A., Calloni G., Torrent Burgas M., Vabulas R.M., De Groot N.S., Tartaglia G.G. (2020). RNA-binding and prion domains: the Yin and Yang of phase separation. Nucleic Acids Res..

[28] Laughery M.F., Hunter T., Brown A., Hoopes J., Ostbye T., Shumaker T., Wyrick J.J. (2015). New vectors for simple and streamlined CRISPR-Cas9 genome editing in Saccharomyces cerevisiae. Yeast.

[29] Tian S., Curnutte H.A., Trcek T. (2020). RNA Granules: A View from the RNA Perspective. Molecules.

[30] Mateju D., Eichenberger B., Voigt F., Eglinger J., Roth G., Chao J.A. (2020). Single-Molecule Imaging Reveals Translation of mRNAs Localized to Stress Granules. Cell.

[31] Davidson A., Parton R.M., Rabouille C., Weil T.T., Davis I. (2016). Localized Translation of gurken/TGF-α mRNA during Axis Specification Is Controlled by Access to Orb/CPEB on Processing Bodies. Cell Rep..

[32] Ray D., Laverty K.U., Jolma A., Nie K., Samson R., Pour S.E., Tam C.L., von Krosigk N., Nabeel-Shah S., Albu M. (2023). RNA-binding proteins that lack canonical RNA-binding domains are rarely sequence-specific. Sci. Rep..

[33] Weinberg J.S., Drubin D.G. (2014). Regulation of Clathrin-Mediated Endocytosis by Dynamic Ubiquitination and Deubiquitination. Curr. Biol..

[34] Dores M.R., Schnell J.D., Maldonado-Baez L., Wendland B., Hicke L. (2010). The function of yeast Epsin and Ede1 ubiquitin-binding domains during receptor internalization. Traffic.

[35] Swaney D.L., Beltrao P., Starita L., Guo A., Rush J., Fields S., Krogan N.J., Villén J. (2013). Global analysis of phosphorylation and ubiquitylation cross-talk in protein degradation. Nat. Methods.

[36] MacGurn J.A., Hsu P.C., Smolka M.B., Emr S.D. (2011). TORC1 regulates endocytosis via Npr1-mediated phosphoinhibition of a ubiquitin ligase adaptor. Cell.

[37] Shchepachev V., Bresson S., Spanos C., Petfalski E., Fischer L., Rappsilber J., Tollervey D. (2019). Defining the RNA interactome by total RNA-associated protein purification. Mol. Syst. Biol..

[38] Casolari J.M., Thompson M.A., Salzman J., Champion L.M., Moerner W.E., Brown P.O. (2012). Widespread mRNA association with cytoskeletal motor proteins and identification and dynamics of myosin-associated mRNAs in S. cerevisiae. PLoS One.

[39] Duran-Arqué B., Cañete M., Castellazzi C.L., Bartomeu A., Ferrer-Caelles A., Reina O., Caballé A., Gay M., Arauz-Garofalo G., Belloc E., Mendez R. (2022). Comparative analyses of vertebrate CPEB proteins define two subfamilies with coordinated yet distinct functions in post-transcriptional gene regulation. Genome Biol..

[40] UniProt Consortium (2023). UniProt: the Universal Protein Knowledgebase in 2023. Nucleic Acids Res..

[41] Baltz A.G., Munschauer M., Schwanhäusser B., Vasile A., Murakawa Y., Schueler M., Youngs N., Penfold-Brown D., Drew K., Milek M. (2012). The mRNA-Bound Proteome and Its Global Occupancy Profile on Protein-Coding Transcripts. Mol. Cell.

[42] Castello A., Fischer B., Eichelbaum K., Horos R., Beckmann B.M., Strein C., Davey N.E., Humphreys D.T., Preiss T., Steinmetz L.M. (2012). Insights into RNA Biology from an Atlas of Mammalian mRNA-Binding Proteins. Cell.

[43] Wang Y., Zhang C., Yang W., Shao S., Xu X., Sun Y., Li P., Liang L., Wu C. (2021). LIMD1 phase separation contributes to cellular mechanics and durotaxis by regulating focal adhesion dynamics in response to force. Dev. Cell.

