# Supplementary material for: Proteasome assembly chaperone translation upon stress requires Ede1 phase separation at the plasma membrane
Source: iScience. 2023 Dec 14;27(1):108732. doi: 10.1016/j.isci.2023.108732 (PMC10792233; doi:10.1016/j.isci.2023.108732)
Supplement: Document S1. Figures S1–S3 and Table S1 [file mmc1.pdf]

iScience, Volume *27*

Supplemental information

Proteasome assembly chaperone translation upon stress requires Ede1 phase separation at the plasma membrane

Thomas D. Williams, Aurellia Winaya, Ifeoluwapo Joshua, and Adrien Rousseau

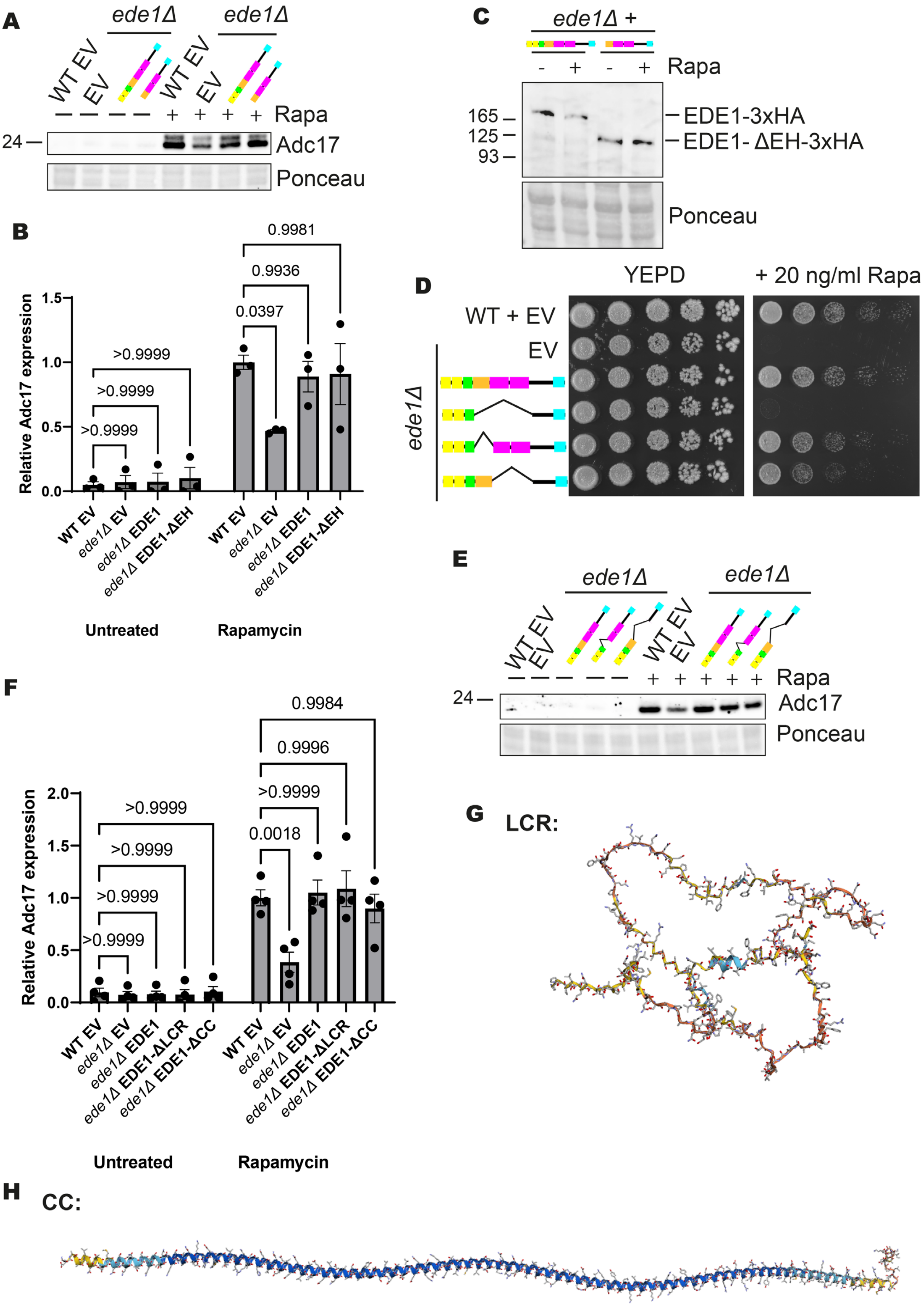

Figure S1: Supporting information for figure 1

Figure S1: Supporting information for figure 1 – analysis of requirement of Ede1 domains for rapamycin resistance and Adc17 expression.
A) Adc17 expression levels in untreated and rapamycin treated WT + empty vector and ede1Δ cells rescued with empty vector, full length Ede1 and Ede1 lacking the N-terminal EH domains.
B) Quantification of A (n=3), showing mean +/- SEM.
C) Expression levels of Ede1-3xHA expressed from the Ede1 promoter in ede1Δ cells (full length and lacking the N-terminal EH domains) in untreated and rapamycin treated conditions.
D) Growth of WT + empty vector and ede1Δ cells rescued with Ede1 (full length, ΔLCR/CC, ΔLCR, ΔCC) after 3 days on YEPD plates +/- rapamycin.
E) Adc17 expression levels in untreated and rapamycin treated WT + empty vector and ede1Δ cells rescued with empty vector, full length Ede1 and Ede1 lacking the LCR or CC domains.
F) Quantification of F (n=4), showing mean +/- SEM.
G) ColabFold prediction of the EDE1 LCR domain structure. Colours denote confidence in the structure with bluer colours being more confident and yellow less confident.
H) ColabFold prediction of the EDE1 CC domain structure. Colours denote confidence in the structure with

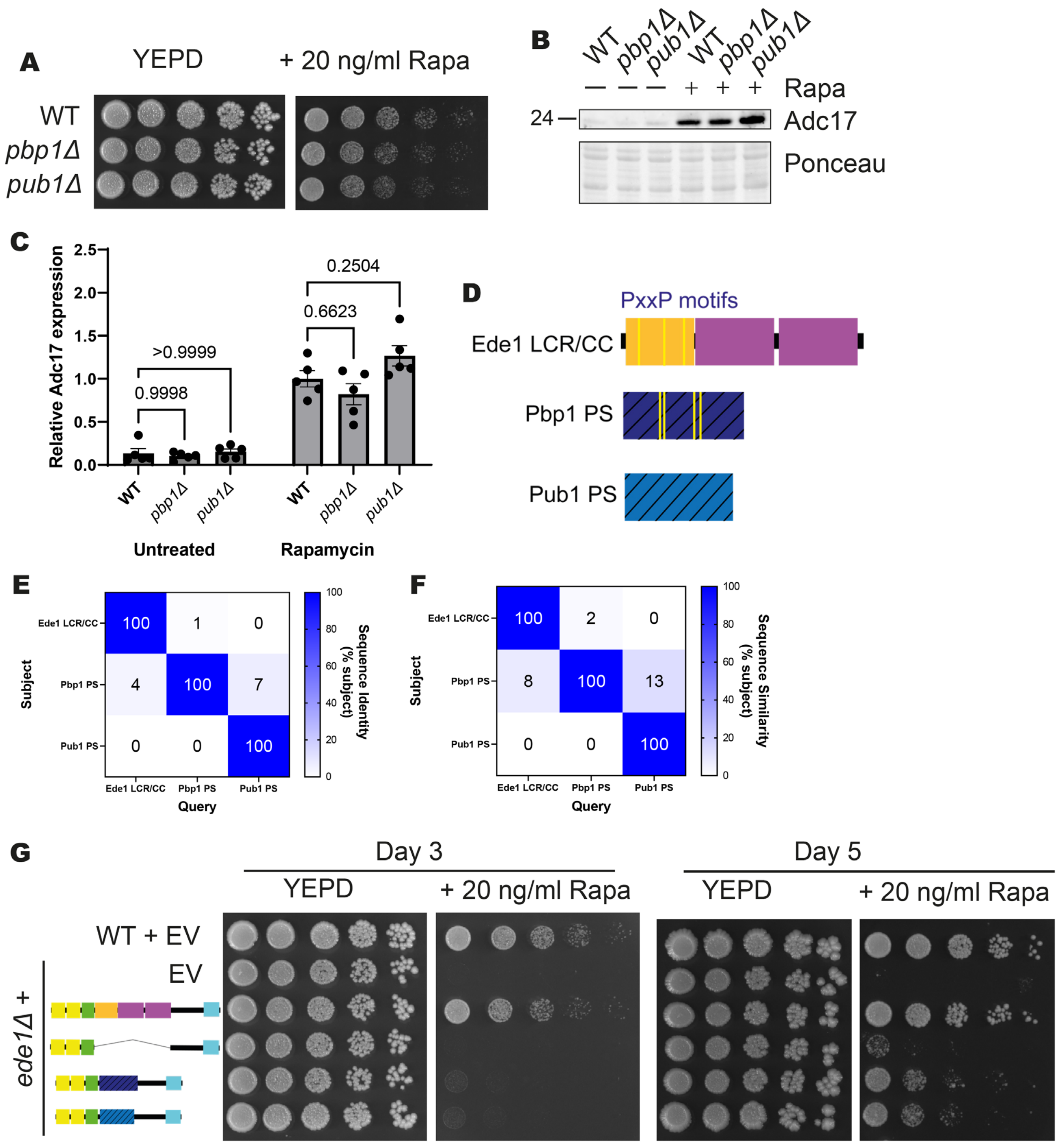

Figure S2: Supporting information for figure 3 – comparison of Pbp1 and Pub1 with Ede1, and their impact on Adc17 expression.
A) Growth of WT, pbp1Δ, and pub1Δ cells after 3 days on YEPD plates +/- rapamycin.
B) Adc17 expression levels in untreated and rapamycin treated pbp1Δ and pub1Δ cells.
C) Quantification of B (n=5), showing mean +/- SEM.
D) PxxP motifs in the Ede1 LCR/CC domains and Pbp1/Pub1 phase separating domains.
E) 3-way comparison of sequence identity between the Ede1 LCR/CC domains and Pbp1/Pub1 phase separating (PS) domains.
F) 3-way comparison of sequence similarity between the Ede1 LCR/CC domains and Pbp1/Pub1 PS domains.
G) Growth of ede1Δ cells rescued with Ede1 (full length, ΔLCR/CC, ΔLCR/CC + Pbp1 PS, and ΔLCR/CC + Pub1 PS) after 3 and 5 days on YEPD plates +/- rapamycin.

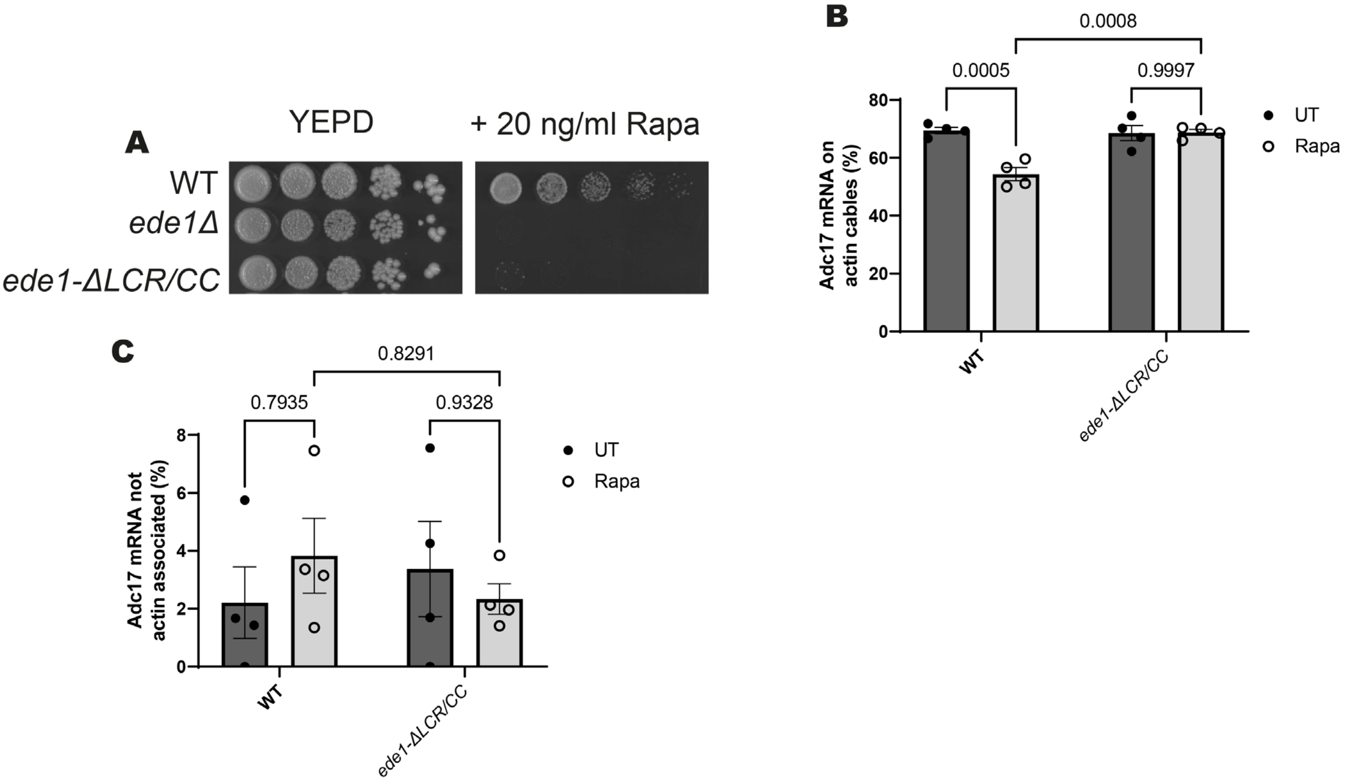

Figure S3: supporting information for figure 4 – Ede1 LCR/CC domains role in ADC17 mRNA localisation.
A) Growth of WT, ede1Δ, and ede1-ΔLCR/CC cells after 3 days on YEPD plates +/- rapamycin.
B) Proportion of ADC17 mRNA localised to actin cables in untreated and 1 h rapamycin treated cells with WT and ΔLCR/CC Ede1 (n=4), showing mean +/- SEM.
C) Proportion of ADC17 mRNA not associated with actin in untreated and 1 h rapamycin treated cells with WT and ΔLCR/CC Ede1 (n=4), showing mean +/- SEM.

Table S1: Oligonucleotides used in this study.

REAGENT or RESOURCE	SOURCE	IDENTIFIER
Oligonucleotides		
GTATTTTTTTACGTAAGAATAATATAATAGCATGACG CTGACGTGTGATTTCGATGAATTCGAGCTCG	Sigma-Aldrich	Abp1-mK2-KI_F
AAGACGGCTCAAAAGGTCTCTTCCCCAGCAATTATG TGTCTTTGGGCAACGGTGACGGTGCTGGTTTA	Sigma-Aldrich	Abp1-mK2-KI_R
ACTCGATCCCAGCTCCAA	Sigma-Aldrich	Abp1-mK2-scrn_A
AGGAAAGCACCATGTACCAT	Sigma-Aldrich	Abp1-mK2-scrn_D
TGCCTTGGAAAAGTGCAACT	Sigma-Aldrich	Ede1-8xHIS gRNA
TGAAGAAGAAGCGCACAATGCCTTGGAAAAGTGCA ACTGGcATCTAGAAGCCGCCACTAACTTTTTGTTGG ATAGTGCTCATCACCACCATCATCACCAT	Sigma-Aldrich	Ede1-8xHIS repair DNA_F
AGAAGTACAAAAAGAAGACGAAATGGTCCATTACAG ACTACTTGTCATCGTCATCTTTATAATCTGCGGCTGC GGCGTGATGGTGATGATGGTGGTGATG	Sigma-Aldrich	Ede1-8xHIS repair DNA_R
GACTTATGCCCTGAACGAGT	Sigma-Aldrich	Ede1-8xHIS-scrn_A
GTCAACCAGTGACCCTGCT	Sigma-Aldrich	Ede1-8xHIS-scrn_D
GGGTCGCTCTTGACTTTTTG	Sigma-Aldrich	Ede1-ΔLCR/CC gRNA
TTTGGTTGTTGTATCTGTATTAGAGGCGCTTTTCGCA ATAATGTCCTTAGCATCCTCAGGTAATTGTCCCATA GCATAAAGTCCTAAAGC	Sigma-Aldrich	Ede1-ΔLCR/CC repair DNA_F
AAAAAGAATGCTGGTGTCGAATTACCGGATGTTATC CCCAATGAATTATTACAGTCCCCCGCTTTAGGACTT TATGCTATGGGACAATTA	Sigma-Aldrich	Ede1-ΔLCR/CC repair DNA_R
CTGACTGGGTGCCGTTGTTTG	Sigma-Aldrich	Ede1-ΔLCR/CC-scrn_A
GAGTGCTAACTCCACTGGCGT	Sigma-Aldrich	Ede1-ΔLCR/CC-scrn_D
